# Mr. George Bell on Excision in Cases of Wounded Nerves

**Published:** 1826-07

**Authors:** 


### WOUNDED NERVES.*

Affections of the nerves have, as Mr. G. Bell truly observes, been always interesting and puzzling too, both to surgeons and physicians, from their anomalous character, obstinate nature, and sometimes fatal consequences. The anatomical labours, however, of Wrisberg, Monro, and Reil, and the physiological researches of Bichat, Home, Scarpa, and last, not least, Charles Bell, have done much, very much, to dispel the darkness which hung over the nervous system, and light the way for clearer views and more certain practice. The errors of our predecessors, nay, of our own times, have been many, but we may now hope for better things. Mr. G. Bell proceeds to state a few cases of injured nerves, and their treatment, with the highly laudable desire of eliciting information from his brother practitioners. But, to the point.

July 7th, 1802, Mr. B. was requested by Dr. James Hamilton to see, with him, a young woman, who had been bled, ten days before, in the median cephalic of the right arm. On examination—the fore-arm was bent to an acute angle with the arm, fingers clenched, and the attempt at

---

* Mr. George Bell. Edinburgh Journal of Medical Science, No. 11.—April.

extension of either the arm or fingers gave excruciating pain—no swelling; lancet-wound healed, but pressure on it exasperates the pain; this was, at times, excessive, shooting down to the tips of the fingers, and upwards to the axilla, clavicle, pectoral muscle, and even short ribs, accompanied by startings, tremors, subsultus tendinum, &c. Pulse 110; considerable fever. As every remedy he could think of had been tried in vain by Dr. H. and as locked-jaw was apprehended, it was determined to excise part of the vein which had been opened, and so make sure of the nerve with it. This was preferred to mere division of the nerve, as there was little chance of hitting on the exact spot of the injury, and the operation would be done at random; accordingly, with the concurrence of Drs. Barclay and Hamilton, an incision was made through the skin, from an inch and a half above, to the same distance below the lancet-wound. The vein being exposed and separated from its connexions, "two ligatures were applied at an inch and a half from each other, and equi-distant from the wound in the vein," the intermediate portion of which was then removed—the tying of the superior ligature was much more painful than that of the lower. The operation was followed by immediate relief to the symptoms; indeed, all she complained of now was the mere uneasiness of the wound: the sides of this were brought together by adhesive plaster, and a pledget, compress and bandage applied. She was then placed in bed, and the arm so laid on a pillow that the flexor muscles were quite relaxed—an opiate was given.

July 8th. She is tolerably easy, and has had a good night, though the fingers were again contracted, most likely from the irritation of the ligatures. She can permit the fingers to be moved, however, in every direction, without increase of pain. 9th, Has passed a restless night—bowels costive—uneasiness in the fingers and fore-arm—emollient poultice to the wound—a dose of pulv. jalap. comp. which acted freely—from this time she continued to improve. On the 12th, the ligatures were removed; and, on the 1st August, she was well, having merely a little numbness and stiffness in the arm, the necessary consequence of the operation.

*Remarks.* The good effects of excision, in this instance, are evident enough; the patient being, in all probability, saved either from tetanus or permanent contraction of the elbow-joint. To shew that an operation may be serviceable, even a considerable time after the infliction of the injury, there is a case given from Volchamer which we shall just glance at.

A young woman, æt. 16, wounded the radial artery and nerve with a knife; the wound healed, leaving only a little pustule. Some months afterwards, being affected with fainting fits, she applied to Volchamer, who, taking the pustule for an incipient aneurism, had caustic applied freely, and the wound kept open for *six months*. The fainting fits did not return.

This instance is rather equivocal; for it is hard to say that the cautery cured the fits, seeing that it was six months about it. But let that pass.

Another case is given by Dr. Milligan, which is rather interesting.

A lady, æt. 22, after being bled in the median basilic of the left arm, complained of pain in the wound, and on going to bed, shortly afterwards, felt great uneasiness in the left shoulder. At three the next morning, she was seized with spasms of the nerves and extensors of the hands; anxiety; increasing pain at the scrobiculus cordis, and even opisthotonos; the muscles of the upper and lower extremities, and the pectorals acting most violently. In about six minutes the spasms went off, but soon returned, and continued to recur for a long period. She took 580 drops of laudanum in the first 24 hours, and in the course of two months not less than 44,000.

Another instance of nervous injury is related by Mr. Bell, where removal of the part was practised.

The lady of a medical man, æt. 26, cut the artery, and probably the nerve, of the thumb, on its radial side, half way between the first and second joints. The wound healed, but she continued to suffer from pain, startings, twitchings of the flexors, contraction of the thumb, and great general irritability. Opiates; every plan was tried without success. On two occasions, incisions were made down to the bone in the neighbourhood of the wound, with but temporary relief. June, 1805, two years after the accident, she consulted Mr. B. Her health had suffered, and the pain and irritability of mind were so great that derangement was to be feared.

Dr. Monro, *secundus*, and Mr. Russel were called in—mercury was tried—it did harm. After the mercurial influence had subsided the thumb was removed at the second joint. In six weeks the lady was well.

Mr. Bell justly observes, that such cases as these will not be confounded with those of inflammation of the veins, or of the cellular tissue as described by Dr. Duncan. With regard to excision, if the case presents early, so much the better, but even after the lapse of months, there is a chance, and *cæteris paribus*, a very fair one, of success. Mr. B. draws a very judicious distinction between the operation here and in tic douloureux, for in the latter affection, we know not whether the disease be in the *root* or in the *extremity* of the nerve, consequently we cut at hazard.

---

